# Value of Plasmatic Membrane Attack Complex as a Marker of Severity in Acute Kidney Injury

**DOI:** 10.1155/2014/361065

**Published:** 2014-05-25

**Authors:** Eva Rodríguez, Marta Riera, Clara Barrios, Julio Pascual

**Affiliations:** ^1^Departamento de Nefrología, Hospital del Mar, Passeig Marítim 25-29, 08003 Barcelona, Spain; ^2^Nefrología Grupo de Investigación, Institut Mar d'Investigacions Médiques, Carrer del Doctor Aiguader 88, 08003 Barcleona, Spain

## Abstract

The aim of this study was to determine if complement pathway is activated in AKI; for this purpose, we measured, through ELISA sandwich, the terminal lytic fraction of the complement system, called membrane attack complex (C5b-C9), in AKI patients compared with patients with similar clinical conditions but normal renal function. Our data showed that complement system is activated in AKI. Plasmatic MAC concentrations were significantly higher in AKI patients than in those with normal renal function; this difference is maintained independently of the AKI etiology and is proportional to the severity of AKI, measured by ADQI classification. In addition, we found that plasmatic MAC concentrations were significantly higher in patients who did not recover renal function at time of hospitalization discharge, in patients who died during the acute process, and in patients who need renal replacement therapy during hospitalization, but in this last group, the differences did not reach statistical significance. In conclusion, plasmatic MAC concentration seems valuable as a marker of AKI severity.

## 1. Introduction

Epidemiological studies have reported that the risk of adverse outcomes is proportional to the severity of acute kidney injury (AKI) [1–4]. Accurate identification of patients with severe renal injury early in the disease course could augment the efficacy of available interventions and improve patient outcomes. However, it is difficult to estimate the severity of AKI at an early time point because AKI staging is based on the magnitude of changes in serum creatinine and urine output, surrogates of glomerular filtration rate (GFR) that do not change until renal injury has occurred [5–7]. The recent Kidney Disease Improving Global Outcomes clinical practice guideline for AKI (K-DIGO) highlighted the need for improved risk assessment for patients with established AKI [8].

Many biomarkers have been proposed as early markers of AKI, which may be useful for the detection of AKI before increases in serum creatinine, neutrophil gelatinase-associated lipocalin (NGAL), kidney injury molecule-1 (KIM-1), IL-18, cystatin C, liver-type fatty acid-binding protein, monocyte chemoattractant protein 1 (MCP-1), prepro-epidermal growth factor (EGF), and urinary components of renin-angiotensin system [9–16]. These biomarkers seem to show different aspects of renal injury; cystatin C concentrations correlate with changes in glomerular filtration rate whereas NGAL concentrations are related to tubular stress or injury [17–20]; urinary EGF excretion was reduced in cisplatin nephrotoxicity, in ischemic kidney injury [21], and after ureteral obstruction supressing tubular apoptosis and enhancing renal tubular cell regeneration [22, 23]. Munshi et al. showed that urinary MCP-1 may be a useful biomarker of AKI, providing the first evidence that urinary histone assessment may be a useful tool in kidney disease [24].

These biomarkers change with treatment or recovery, which suggests that they may be used to monitor interventions [25].

Novel biomarkers increase our understanding of the pathogenesis of AKI by identifying possible mechanisms of injury. Currently NGAL is the most studied renal biomarker and probably the most promising of them because of the results obtained in different scenarios and clinical conditions [26–30].

Complement activation is an important mechanism of renal injury in different diseases affecting each of the renal compartments (glomerulus, tubulointerstitium, and vascular departments) [31]. The complement system is an important innate humoral defense system comprised of more than 20 plasma proteins that may be activated in a cascade fashion by either the classic pathway (immune complex mediated) or the alternative pathway. A regulatory system of both plasma proteins and membrane bound proteins acts to prevent the inappropriate activation of complement by autologous cells [31].

Complement activation has been shown to be an important event in the development of ischemic AKI in mice. Studies in complement-deficient mice have shown that mice are protected from renal failure after ischemia/reperfusion (I/R) [31, 32], and that generation of the anaphylatoxin C5a [33] and the membrane attack complex (C5b-C9 or MAC) [32] may contribute to the pathogenesis of ischemic AKI. The proximal tubule is the primary damaged site after renal I/R; complement activation on the ischemic tubule is an important contributor to ischemic AKI. In addition, treatment with agents that inhibit the complement cascade at specific steps has proven effective at ameliorating ischemic AKI [33, 34]; and therapeutic targeting of classical and lectin pathways protects from ischemia-reperfusion-induced renal damage in animal model of kidney transplantation [35]. There is growing evidence that, in animal model of transplant kidney, complement plays a critical role in the acute induction of endothelial-to -mesenchymal transition, suggesting that therapeutic inhibition may be essential to prevent vascular damage and tissue fibrosis [36].

Complement activation in kidney occurs via the alternative pathway [31] and is independent of natural antibody [37].

Uncontrolled alternative pathway activation within the microvasculature is the primary cause of atypical haemolytic uremic syndrome (aHUS) [38]. The complement is also an important mediator of injury in ANCA-associated vasculitis [39] and antiglomerular basement membrane disease [40].

The MAC forms pores in cells resulting in cell activation. At high concentration, it causes cell death by lysis. Sublytic doses of MAC can activate renal parenchymal cells, which then release proinflammatory cytokines, reactive oxygen species, vasoactive chemicals, and profibrotic factors [41–44].

The aim of this study was to determine if MAC serum concentrations may allow clinicians to identify AKI-patients at high risk of adverse outcomes.

## 2. Material and Methods

### 2.1. Patients

We designed a case-control study that enrolled patients diagnosed with AKI in the Hospital del Mar in Barcelona, Spain, between October 2010 and December 2012. Patients were enrolled if they were at least 18 years of age and ADQI (Acute Dialysis Quality Initiative) serum creatinine criteria were met. Patients with chronic kidney disease were excluded. A control group included patients with the same clinical condition but normal renal function.

Case and control groups were classified according to the main etiology of AKI in four groups: septic group, ischemia-reperfusion group, nephrotoxicity group, and multifactorial group. The septic group included patients admitted in Intensive Care Unit (ICU) with sepsis or septic shock diagnosis with AKI (case group) or normal renal function (control group). Ischemia-reperfusion group included renal allograft recipients with delayed graft function (case group) or immediate good renal function (control group). Nephrotoxicity group included domiciliary hospitalization patients under colistin treatment with AKI (case group) or normal renal function (control group). Colistin is an antibiotic with a well-documented tubular toxicity [45]; domiciliary patients were enrolled, to avoid selection bias between septic and toxicity groups. Finally a multifactorial group was designed including patients with multiple AKI risk factors such as dehydration, contrast administration, and nonsteroidal anti-inflammatory therapy with AKI (case group) or normal renal function (control group).

AKI was defined according to the ADQI (Acute Dialysis Quality Initiative) criteria (RIFLE classification) [15, 16]. Briefly, patients were classified into the “risk” category if serum creatinine increased 1.5-fold, “injury” if serum creatinine increased 2-fold, and “failure” if serum creatinine increased 3-fold. The outcome criteria of loss of renal function and end-stage renal disease (ESRD) were defined by the duration of AKI.

Urine output criteria were not used in diagnosis or staging because these data were not available.

Patients were followed until time of hospital discharge or death and were staged according to the maximum increase in serum creatinine using the RIFLE classification.

For each individual patient, we recorded demographical data, past medical history, and laboratory data on admission and when AKI was solved.

This study adhered to the Principles of Helsinki Declaration, and the hospital's Ethics Committee (CEIC-IMAS) approved the study protocol, and all participants gave their written informed consent to participate in this study.

### 2.2. Plasma Samples and MAC Measurement

Samples were collected at the time of AKI diagnosis, except in ischemia-reperfusion group, in which samples were collected on the seventh day after transplantation, and when AKI was solved.

Whole blood samples were collected in EDTA tubes to prevent further* in vitro* complement activation. Samples were transported on ice and centrifuged to obtain plasma, and plasma was stored in aliquots at –80°C avoiding multiple freeze/thaw cycles.

Plasma samples were thawed at 37°C, and sandwich ELISAs were used to measure C5b-C9 membrane attack complex (MAC) according to the manufacturer's protocol (Hycult biotech). Briefly, samples and standards were incubated in microtiter wells coated with antibodies recognizing human MAC, streptavidin-peroxidase conjugate was used to bind the biotinylated tracer antibody, and the enzyme reaction was stopped by the addition of oxalic acid. The absorbance at 450 nm was measured with a spectrophotometer, a standard curve was obtained by plotting the absorbance versus the corresponding concentrations of the MAC, and finally, the MAC concentrations were determined from the standard curve.

### 2.3. Statistical Analyses

Data are presented as mean (± standard deviation), absolute numbers, or percentages. Statistical significance was evaluated by using the Student's* t*-test or Paired* t*-test when it was required. Multiple-group comparisons were performed using ANOVA test, and univariate receiver-operating characteristic (ROC) curve analysis was performed to determine whether plasmatic MAC concentrations predicted AKI and the predictive value of MAC for AKI. ROC curves were considered statistically significant if the 95% CI of the area under the ROC curve (AUC) did not overlap 0.5. Statistical tests were performed in SPSS software, v 21, Chicago, IL, USA.

## 3. Results

### 3.1. Patient Characteristics

**Figure 1:**
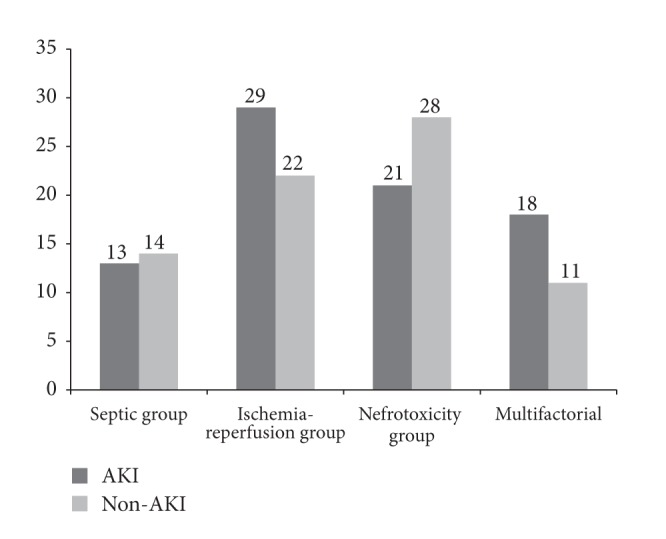
Distribution according to the AKI etiology, expressed in absolute numbers.

A total of 156 patients were included. Eighty one of them (52%) were diagnosed as having AKI and 75 (48%) were controls with normal renal function (non-AKI patients). Distribution according to the main AKI etiology was as follows: septic group (*n* = 27; AKI 13 patients (52%) versus non-AKI 14 patients (48%)), ischemia-reperfusion group (*n* = 51; AKI 29 patients (57%) versus non-AKI 22 patients (43%)), nephrotoxicity group (*n* = 49; AKI 21 patients (43%) versus non-AKI 28 patients (57%)), and multifactorial group (*n* = 29; AKI 18 patients (62%) versus non-AKI 11 patients (38%)). Distribution according to the main etiology is detailed in Figure 1.

All patients were Caucasian, mean age was 61.3 ± 15.2 years, and 102 (65%) were men. There were no statistically significant differences in demographic variables and past medical history data between AKI group and non-AKI group. Baseline characteristics of the study population are shown in Table 1.

For analysis, patients were assigned to their worst RIFLE category according to serum creatinine criteria. Fifteen patients (18.5%) were included in “risk” group; 13 patients (16%) were included in “injury” group; 33 patients (40.7%) were included in “failure” group; 20 patients (24.8%) were included in “loss” group, and nobody was classified in End-Stage-Renal-Disease (ESRD).

During hospitalization, 27 AKI patients needed renal replacement therapy; 21 of them recovered renal function, fully or partially, without the need to hemodialysis at time of discharge. In the AKI group (81 patients), at time of hospital discharge, 60 patients recovered partial or full renal function, 36 (23.1%) and 24 (15.4%), respectively.

### 3.2. Plasmatic Concentrations of C5B-C9 Complement Membrane Attack (MAC)

**Figure 2:**
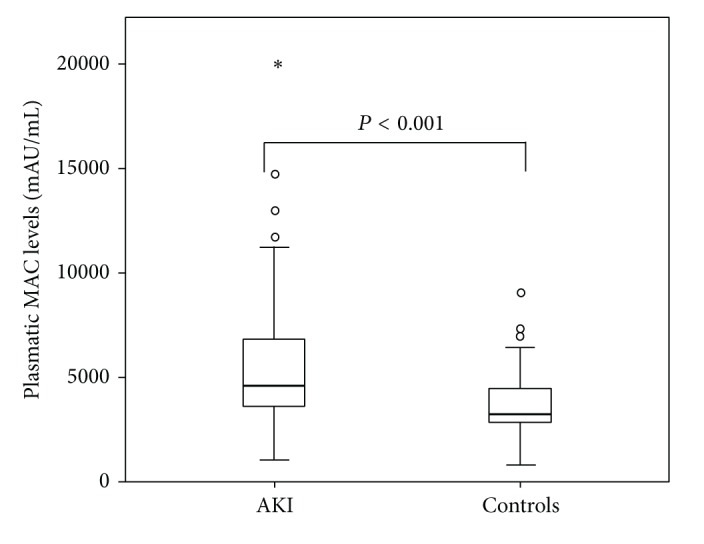
Comparison of MAC plasmatic concentrations between AKI patients and control patients.

Plasmatic MAC concentration was significantly higher in AKI-patients than in those with normal renal function (5848 ± 83 versus 3702 ± 52 mAU/mL, *P* < 0.01) (Figure 2). The difference is maintained independently of the AKI etiology (Table 2) and is proportional to the severity of AKI, measured by RIFLE classification; the mean value of MAC concentrations in “Risk” patients was 4905.4 mAU/mL and in “Injury” patients was 5246.3 mAU/mL, and the mean value of plasmatic MAC in “Failure” patients was 6971 mAU/mL, showing a progressive increase. In addition, correlation coefficient showed a direct relationship between serum creatinine and MAC plasmatic concentrations (Pearson coefficient 0.28, *P* < 0.01).

In AKI-patients, plasmatic MAC concentration showed a significant decrease when AKI was solved (5213 ± 685 versus 3402.5 ± 465 mAU/mL, *P* < 0.02). In contrast, plasmatic MAC of non-AKI patients did not show any change when the acute episode (sepsis, colistin administration, etc.) was solved.

Plasmatic MAC concentration was significantly lower in AKI-patients who recovered renal function, compared with those patients who did not recover renal function at time of hospital discharge (5076.3 ± 277 versus 6146.8 ± 390 mAU/mL, *P* < 0.001).

In AKI-patients who died during hospitalization, we found significantly higher plasmatic MAC concentrations compared with AKI-patients who survived (7026 ± 170 versus 5216 ± 240 mAU/mL, *P* < 0.001).

In contrast, differences in MAC concentrations between AKI-patients who needed renal replacement therapy compared with those who did not need hemodialysis during AKI episode did not reach statistical significance (6158.9 ± 527 versus 5699.2 ± 595 mAU/mL, *P* < 0.07).

**Figure 3:**
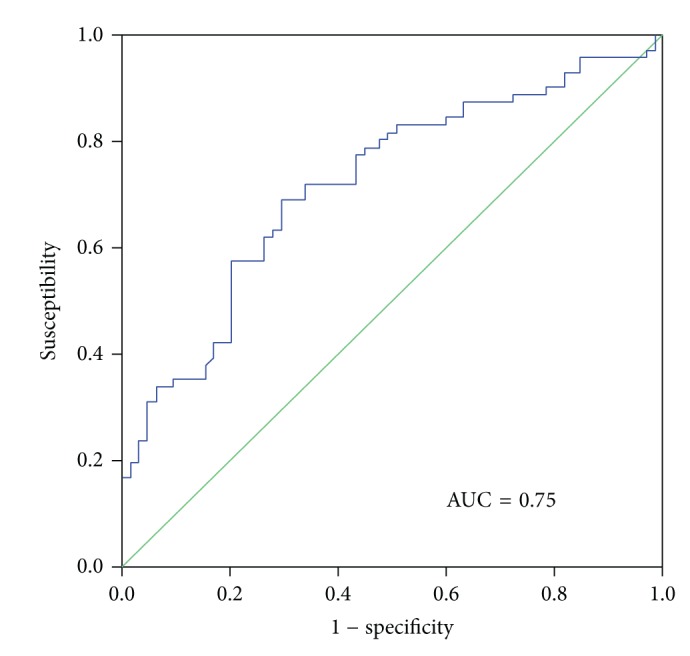
Univariate receiver-operating characteristic (ROC) curves for MAC plasmatic concentrations.

The discrimination value of the plasmatic MAC concentration for AKI diagnosis was established by means of a receiver-operating characteristic (ROC) curve, with an area under the curve (AUC) of 0.75 (95% CI 0.64 to 0.86; *P* < 0.001) (Figure 3), and the cut-off value with the best sensitivity (75%) and specificity (70%) in predicting AKI was established in 3900 mAU/mL, positive predictive value 70%.

## 4. Discussion

Our data showed that complement pathway is activated in AKI, regardless the etiology of AKI, leading to the production of lytic complex C5b-C9 or MAC. Plasmatic MAC concentrations significantly decrease when AKI is solved. In addition, MAC concentrations were directly proportional to the severity of AKI, quantified by RIFLE classification.

These data agree with previous studies that showed complement activation as an important mechanism of renal injury in different diseases, aforementioned, aHUS [38], renal injury in ANCA-associated vasculitis [39], and antiglomerular basement membrane disease [40]. The proximal tubule is the primary site of injury after renal I/R, and complement activation on the ischemic tubule is an important cause of ischemic AKI [31]. C6-deficient mice, unable of generating MAC, are protected from renal I/R [32]. Given that complement activation is most prominent in the tubulointerstitium, the primary targets of MAC are likely tubular epithelial cells. MAC formation on the cell surface could contribute to cell necrosis, exacerbate adenosine triphosphate depletion (ATP) in hypoxic cells [46], and trigger intracellular signaling pathways and cell activation [47]. Fewer neutrophils infiltrated the kidneys in C6 deficient mice after I/R [32], which may be a direct effect of reduced inflammatory signaling. When complement is activated on cell or tissue surfaces, some of the formed MAC remains soluble (cytolytically inactive) and can increase adhesion molecule expression and promote neutrophil infiltration [48, 49] providing another potential link between complement activation on the tubular epithelial cells and renal inflammation.

In our study, plasmatic MAC concentrations identify AKI patients at risk of developing serious outcomes like death during hospitalization or unrecovered renal function at time of hospital discharge. Plasmatic MAC concentrations were high in AKI patients who needed renal replacement therapy, but these differences did not reach statistical significance, probably due to sample size.

The discrimination value of MAC concentrations was established by means of a ROC curve, and our results demonstrated that MAC AUC was not very different to that previously observed in NGAL studies [50].

Our study has several limitations. It is retrospective in nature, despite the fact that serum samples were prospectively collected. In our hospital there is not any important Cardiac Surgery Department, and the I/R human model used was a renal allograft transplantation, which may add some immunological factors to the I/R derived injury. However, our results were consistent across the 4 types of AKI assessed.

In conclusion, plasmatic MAC concentration seems valuable as a marker of severity in AKI patients of different etiologies. Larger studies are needed to delineate its true power of discrimination.

## Figures and Tables

**Table 1:** Baseline characteristics of study population.

	AKI (*n* = 81)	Non-AKI (*n* = 75)
Age (years)	64.3 ± 14.3	61.5 ± 16.4
Male sex	50 (61.7%)	52 (69.3%)
Diabetes mellitus	21 (25.9%)	14 (18.7%)
Hypertension	55 (67.9%)	44 (58.7%)
Coronary artery disease	6 (7.5%)	16 (21.3%)
Mortality	17 (21%)	11 (14.7%)

AKI: Acute kidney injury.

**Table 2:** Plasmatic membrane attack complex concentrations and etiologies.

AKI Etiology	AKI	Non-AKI	*P*
Septic group	5407.5 ± 997.4	3743.4 ± 408.3	0.001
Ischemia-reperfusion group	8040 ± 816.4	4450.6 ± 382.7	0.001
Nephrotoxicity group	4906.6 ± 636.5	1278.7 ± 152.3	0.002
Multifactorial group	4720.6 ± 257	2312.3 ± 236.4	0.001

AKI: Acute kidney injury.

## References

[1] Chertow GM, Burdick E, Honour M, Bonventre JV, Bates DW (2005). Acute kidney injury, mortality, length of stay, and costs in hospitalized patients. *Journal of the American Society of Nephrology*.

[2] Ricci Z, Cruz D, Ronco C (2008). The RIFLE criteria and mortality in acute kidney injury: a systematic review. *Kidney International*.

[3] Zhou J, Yang L, Zhang K, Liu Y, Fu P (2012). Risk factors for the prognosis of acute kidney injury under the acute kidney injury network definition: a retrospective, multicenter study in critically ill patients. *Nephrology*.

[4] Uchino S, Bellomo R, Goldsmith D, Bates S, Ronco C (2006). An assessment of the RIFLE criteria for acute renal failure in hospitalized patients. *Critical Care Medicine*.

[5] Bellomo R, Ronco C, Kellum JA, Mehta RL, Palevsky P (2004). Acute renal failure—definition, outcome measures, animal models, fluid therapy and information technology needs: the Second International Consensus Conference of the Acute Dialysis Quality Initiative (ADQI) Group. *Critical Care*.

[6] Mehta RL, Kellum JA, Shah SV (2007). Acute kidney injury network: report of an initiative to improve outcomes in acute kidney injury. *Critical Care*.

[7] Murray PT, Devarajan P, Levey AS (2008). A framework and key research questions in AKI diagnosis and staging in different environments. *Clinical Journal of the American Society of Nephrology*.

[8] Mishra J, Qing MA, Prada A (2003). Identification of neutrophil gelatinase-associated lipocalin as a novel early urinary biomarker for ischemic renal injury. *Journal of the American Society of Nephrology*.

[9] Mishra J, Dent C, Tarabishi R (2005). Neutrophil gelatinase-associated lipocalin (NGAL) as a biomarker for acute renal injury after cardiac surgery. *The Lancet*.

[10] Han WK, Bailly V, Abichandani R, Thadhani R, Bonventre JV (2002). Kidney injury molecule-1 (KIM-1): a novel biomarker for human renal proximal tubule injury. *Kidney International*.

[11] Parikh CR, Jani A, Melnikov VY, Faubel S, Edelstein CL (2004). Urinary interleukin-18 Is a marker of human acute tubular necrosis. *American Journal of Kidney Diseases*.

[12] Herget-Rosenthal S, Marggraf G, Husing J (2004). Early detection of acute renal failure by serum cystatin C. *Kidney International*.

[13] Portilla D, Dent C, Sugaya T (2008). Liver fatty acid-binding protein as a biomarker of acute kidney injury after cardiac surgery. *Kidney International*.

[14] Alge JL, Karakala N, Neely BA (2013). Association of elevated urinary concentration of renin-angiotensin system components and severe AKI. *Clinical Journal of the American Society of Nephrology*.

[15] Alge JL, Karakalan N, Neely BA (2013). Urinary angiotensinogen and risk of severe AKI. *Clinical Journal of the American Society of Nephrology*.

[16] Alge JL, Karakala N, Neely BA, Janech MG, Velez JC, Arthur JM (2013). Urinary angiotensinogen predicts adverse outcomes among acute kidney injury patients in the intensive care unit. *Critical Care*.

[17] Koyner JL, Bennett MR, Worcester EM (2008). Urinary cystatin C as an early biomarker of acute kidney injury following adult cardiothoracic surgery. *Kidney International*.

[18] Devarajan P (2010). Review: neutrophil gelatinase-associated lipocalin: a troponin-like biomarker for human acute kidney injury. *Nephrology*.

[19] Bachorzewska-Gajewska H, Malyszko J, Sitniewska E, Malyszko JS, Dobrzycki S (2006). Neutrophil-gelatinase-associated lipocalin and renal function after percutaneous coronary interventions. *American Journal of Nephrology*.

[20] Haase M, Bellomo R, Devarajan P, Schlattmann P, Haase-Fielitz A (2009). Accuracy of neutrophil gelatinase-associated lipocalin (NGAL) in diagnosis and prognosis in acute kidney injury disease: a systematic review and meta-analyses. *American Journal of Kidney Diseases*.

[21] Safirstein R, Zelent AZ, Price PM (1989). Reduced renal prepro-epidermal growth factor mRNA and decreased EGF excretion in ARF. *Kidney International*.

[22] Kennedy WA, Buttyan R, García-Montes  E, Agati V D, Olsson CA, Sawczuk IS (1997). Epidermal growth factor suppresses renal tubular apoptosis following ureteral obstruction. *Urology*.

[23] Humes HD, Cieslinski DA, Coimbra TM, Messana JM, Galvao C (1989). Epidermal growth factor enhances renal tubule cell regeneration and repair and accelerates the recovery of renal function in postischemic acute renal failure. *Journal of Clinical Investigation*.

[24] Munshi R, Johnson A, Siew ED (2011). MCP-1 gene activation marks acute kidney injury. *Journal of the American Society of Nephrology*.

[25] Srisawat N, Wen X, Lee M (2011). Urinary biomarkers and renal recovery in critically ill patients with renal support. *Clinical Journal of the American Society of Nephrology*.

[26] Haase M, Devarajan P, Haase-Fielitz A (2011). The outcome of neutrophil gelatinase-associated lipocalin-positive subclinical acute kidney injury: a multicenter pooled analysis of prospective studies. *Journal of the American College of Cardiology*.

[27] Parikh CR, Garg AX (2009). Testing new biomarkers for acute kidney injury: association, prediction, and intervention. *American Journal of Kidney Diseases*.

[28] Cruz DN, de Cal M, Garzotto F (2010). Plasma neutrophil gelatinase-associated lipocalin is an early biomarker for acute kidney injury in an adult ICU population. *Intensive Care Medicine*.

[29] Bagshaw SM, Bennett M, Haase M (2010). Plasma and urine neutrophil gelatinase-associated lipocalin in septic versus non-septic acute kidney injury in critical illness. *Intensive Care Medicine*.

[30] Nickolas TL, O’Rourke  MJ, Yang J (2008). Sensitivity and specificity of a single emergency department measurement of urinary neutrophil gelatinase-associated lipocalin for diagnosing acute kidney injury. *Annals of Internal Medicine*.

[31] Thurman JM, Ljubanovic D, Edelstein CL, Gilkeson GS, Holers VM (2003). Lack of a functional alternative complement pathway ameliorates ischemic acute renal failure in mice. *Journal of Immunology*.

[32] Zhou W, Farrar CA, Abe K (2000). Predominant role for C5b-9 in renal ischemia/reperfusion injury. *Journal of Clinical Investigation*.

[33] de Vries B, Köhl J, Leclercq WKG (2003). Complement factor C5a mediates renal ischemia-reperfusion injury independent from neutrophils. *Journal of Immunology*.

[34] Pratt JR, Jones ME, Dong J (2003). Nontransgenic hyperexpression of a complement regulator in donor kidney modulates transplant ischemia/reperfusion damage, acute rejection, and chronic nephropathy. *The American Journal of Pathology*.

[35] Castellano G, Melchiorre R, Loverre A (2010). Therapeutic targeting of classical and lectin pathways of complement protects from ischemia-reperfusion-induced renal damage. *The American Journal of Pathology*.

[36] Curci C, Castellano G, Stasi A (2014). Endothelial-to-mesenchymal transition and renal fibrosis in ischaemia/reperfusion injury are mediated by complement anaphylatoxins and Akt pathway. *Nephrology Dialysis Transplantation*.

[37] Park P, Haas M, Cunningham PN, Bao L, Alexander JJ, Quigg RJ (2002). Injury in renal ischemia-reperfusion is independent from immunoglobulins and T lymphocytes. *American Journal of Physiology: Renal Physiology*.

[38] Noris M, Mescia F, Remuzzi G (2012). STEC-HUS, atypical HUS and TTP are all diseases od complement activation. *Nature Reviews: Nephrology*.

[39] Xiao H, Schreiber A, Heeringa P, Falk RJ, Jennette JC (2007). Alternative complement pathway in the pathogenesis of disease mediated by anti-neutrophil cytoplasmic autoantibodies. *The American Journal of Pathology*.

[40] Quigg RJ, He C, Lim A (1998). Transgenic mice overexpressing the complement inhibitor crry as a soluble protein are protected from antibody-induced glomerular injury. *The Journal of Experimental Medicine*.

[41] Biancone S, Caserta C (1997). Alternative pathway complement activation induces proinflammatory activity in human proximal tubular epithelial cells. *Nephrology, Dialysis, Transplantation*.

[42] Biancone L, David S, Della Pietra V, Montrucchio G, Cambi V, Camussi G (1994). Alternative pathway activation of complement by cultured human proximal tubular epithelial cells. *Kidney International*.

[43] Campbell AK, Morgan BP (1985). Monoclonal antibodies demonstrate protection of polymorphonuclear leukocytes against complement attack. *Nature*.

[44] Takano T, Cybulsky AV (2000). Complement C5b-9-mediated arachidonic acid metabolism in glomerular epithelial cells: role of cyclooxygenase-1 and-2. *The American Journal of Pathology*.

[45] Shavit L, Manilov R, Wiener-Well Y, Algur N, Slotki I (2013). Urinary neutrophil gelatinase associated lipocalin for early detection of acute kidney injury in geriatric patients with urinary tract infection treated by colistin. *Clinical Nephrology*.

[46] Papadimitriou JC, Ramm LE, Drachenberg CB, Trump BF, Shin ML (1991). Quantitative analysis of adenine nucleotides during the prelytic phase of cell death mediated by C5b-9. *Journal of Immunology*.

[47] Buerger A, Wagner C, Hug F, Hansch GM (1999). Up-regulation of intracellular calcium, cyclic adenosine monophosphate and fibronectin synthesis in tubuar epithelial cells by complement. *European Journal of Immunology*.

[48] Dobrina A, Pausa M, Fischetti F (2002). Cytolytically inactive terminal complement complex causes transendothelial migration of polymorphonuclear leukocytes in vitro and in vivo. *Blood*.

[49] Tedesco F, Pausa M, Nardon E, Introna M, Mantovani A, Dobrina A (1997). The cytolytically inactive terminal complement complex activates endotelial cells to express adhesion molecules and tissue factor procoagulant activity. *The Journal of Experimental Medicine*.

[50] Flo TH, Smith KD, Sato S (2004). Lipocalin 2 mediates an innate immune response to bacterial infection by sequestrating iron. *Nature*.

